# Distribution and neuronal circuit of spexin 1/2 neurons in the zebrafish CNS

**DOI:** 10.1038/s41598-019-41431-7

**Published:** 2019-03-22

**Authors:** Eunmi Kim, Inyoung Jeong, Ah-Young Chung, Suhyun Kim, Seung-Hae Kwon, Jae Young Seong, Hae-Chul Park

**Affiliations:** 1 Department of Biomedical Sciences, Korea University, Ansan, Gyeonggido 425-707 Republic of Korea; 2 Korea Basic Science Institute, Chun-Cheon, Gangwon-Do 24341 Republic of Korea; 3 Department of Biomedical Sciences, Korea University, Seoul, 136-705 Republic of Korea

## Abstract

Spexin (SPX) is a highly conserved neuropeptide that is widely expressed in mammalian brain and peripheral tissue. In teleost, SPX1 is mainly expressed in the brain and ovary, and is involved in reproduction and food intake. A second form of SPX, SPX2, was recently identified in chick, Xenopus, and zebrafish. The expression pattern and roles of SPX2 are unknown. SPX (*spx*1) is highly expressed in the vertebrate brain, but its distribution, circuits, and interactions with its putative receptor are unknown. Here, we observed expression of *spx1* in the midbrain and hindbrain, and *spx*2 in the hypothalamic preoptic area using *in situ* RNA hybridization in zebrafish. Analysis of transgenic reporter zebrafish revealed that hindbrain SPX1 neurons are PAX2^+^ inhibitory interneurons and project to the spinal cord, where they interact with galanin receptor 2b (GALR2b) neurons, suggesting that hindbrain SPX1 neurons are reticulospinal neurons. *spx*1 mRNA and SPX1 reporter expression were observed in dorsal habenula (dHb). SPX1 neurons in the dHb project to the interpeduncular nucleus (IPN), where GALR2a and GALR2b expression was also observed, suggesting that habenula SPX1 neurons may interact with GALR2a/2b in the IPN.

## Introduction

Neuropeptides play an important role in physiological functions, such as sleep, learning, memory, food intake, and regulation of body temperature. Neuropeptides act through a large variety of G-protein-coupled receptors (GPCRs) which are expressed on the cell surface, thereby contributing to neuronal communication. Spexin (also known as SPX, NPQ, and C12ORF39) is a secreted neuropeptide identified using bioinformatics^1,2^. SPX consists of 14 amino acids that are highly conserved across vertebrate species, including human, rat, and goldfish. Previous reports have shown that the mammalian SPX is widely expressed in numerous tissues, including brain, pancreas, kidney^1–3^, and adrenal gland^4^. RT-PCR analysis in teleosts such as goldfish and zebrafish has revealed that SPX (also known as SPX1) is expressed in the brain, ovary, liver, intestine, kidney, and heart^5,6^.

The diversity of SPX expression suggests that SPX has multiple physiological functions. For example, SPX induces stomach muscle contraction^1^ and inhibits adrenocortical cell proliferation^4^ in rat. In mice, SPX increases arterial pressure, decreases heart rate and urine flow, and has antinociceptive activity^7^. SPX is also associated with obesity and metabolism^8–10^. In goldfish, SPX1 suppresses luteinizing hormone (LH) in the reproductive axis^5^ and reduces food consumption^6^. However, *spx1* knock-out zebrafish show normal reproductive capability but higher food intake than that of wildtype fish, indicating that SPX1 is involved in the control of feeding but not reproduction in zebrafish^11^. Recently, a second form of SPX, namely SPX2, has been identified in chick, Xenopus, and fish models using bioinformatics^12^. However, the expression pattern and physiological roles of SPX2 have yet to be elucidated.

A recent ligand-receptor interaction study reported that SPX1/2 bind human galanin receptor type 2 (GALR2) and type 3 (GALR3), with higher potency toward GALR3 than that of galanin. Moreover, a recent study demonstrates that SPX, unlike galanin, is a biased agonist toward GALR2 preferring G-protein-mediated signalling over β-arrestin-dependent pathways, leading to signalling outputs which are different from those by galanin^13^. There are two forms of GALR2 in zebrafish, GALR2a and GALR2b; GALR3 has not been identified in this species. Zebrafish SPX1/2 also bind GALR2a/2b with higher potency toward GALR2b than that of galanin^12^, indicating that SPX1/2 are functional agonists for zebrafish GALR2b. However, evidence for a direct interaction of SPX1/2 neurons with GALR2/3-epxressing cells *in vivo* has not been reported.

To assess the functions of SPX1/2 and interaction of SPX1 with its putative receptor, GALR2b, in zebrafish, we first investigated the expression of *spx1* and *spx*2 in the developing and adult zebrafish central nervous system (CNS) by whole-mount *in situ* RNA hybridization. We next generated a transgenic reporter zebrafish line, Tg(*spx1:mCherryCAAX*) and *Tg*(*spx1:gal4*; *uas:egfp*), and analysed the distribution of SPX1 neurons and their interaction with GALR2b in the larvae and adult zebrafish CNS. Our findings provide evidence for potential functions of SPX1 and SPX2 in the zebrafish CNS.

## Results

### Distinct expression of *spx1* and *spx2* in the brain of zebrafish larvae

**Figure 1:**
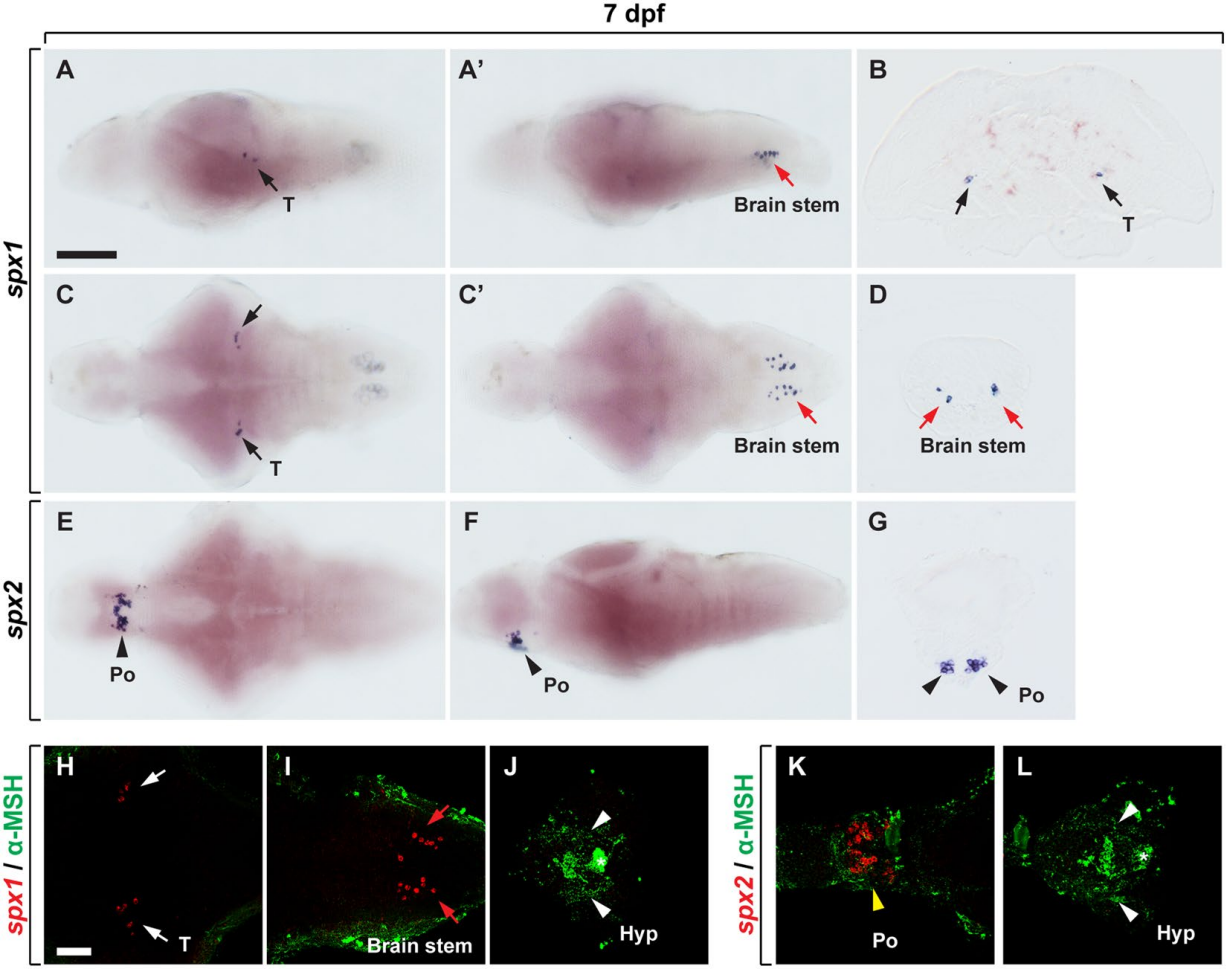
Expression of *spx 1* and *spx 2* mRNA in the brain of zebrafish larvae. (**A**–**G**) *In situ* RNA hybridization with *spx1* (**A**–**D**) and *spx2* RNA probes (**E**–**G**) in the brain of zebrafish larvae at 7 days post-fertilisation (dpf). Lateral (**A**,**A’**,**F**), dorsal (**C**,**C’**), and ventral views (**E**) of the brain, anterior to the left. Transverse sections of the brain (**B**,**D**,**G**), dorsal to the top. (**A**–**D**) *spx1* expression in the midbrain (**A**–**C**) and hindbrain (**A’**,**C’**,**D**). Black and red arrows indicate *spx1* expression in the midbrain tegmentum and hindbrain, respectively. (**E**–**G**) *spx2* mRNA expression in the preoptic area of the hypothalamus. Arrowheads indicate *spx2* expressing cells. (**E**–**G**,**K**,**L**). (**H**–**L**) Labelling with anti-α-melanocyte stimulating hormone (α-MSH) antibody following *in situ* RNA hybridization with *spx1* (**H**–**J**) and *spx2* (**K**,**L**). Ventral views with anterior to the left. White and red arrows indicate *spx1*-expressing cells in the midbrain (**H**) and hindbrain (**I**), and yellow arrowhead indicates *spx2*-expressing cells in the preoptic area (**K**). White arrowheads and asterisk label the hypothalamus and pituitary gland, respectively. Abbreviation: Hyp, hypothalamus; Po, preoptic region; T, midbrain tegmentum. Scale bar: 100 μm in A,A’,C,C’,E,F,E; 50 μm in B,D,G–L.

To examine the expression pattern of *spx1* and *spx2* during development, we first cloned *spx1* and *spx2* cDNA and analyzed their expression by RT-PCR. *spx1* mRNA was first detected in one-cell stage zebrafish embryo but the expression faded, and then reappeared at 24 hpf. *spx2* expression was detected from one-cell stage to 48 hpf continuously (Supplementary Fig. 1A). Whole-mount *in situ* RNA hybridization revealed that *spx1* expression is first detected in a few cells in the hindbrain at 24 hpf (Supplementary Fig. 1B,C), and its expression was detected in the midbrain tegmentum and hindbrain of zebrafish larvae (Fig. 1A–D). However, we did not detect *spx1* expression outside the brain. Conversely, *spx2* mRNA was not detectable during development by whole-mount *in situ* RNA hybridization, but its expression was detected in the preoptic area of the hypothalamus of larval brain (Fig. 1E–G). We think that *spx2* expression level is too low to be detected by whole-mount *in situ* RNA hybridization during development. The distinct expression pattern of *spx1* and *spx2* in the zebrafish brain suggests that the functions of different forms of SPX may be specialised or sub-functionalized in zebrafish. To confirm the expression of *spx1* and *spx2* in the hypothalamus, where SPX1 is involved in reproduction and feeding control in teleost^5,11^, we performed fluorescent *in situ* RNA hybridization for *spx1* or *spx2* followed by immunohistochemistry with an antibody against α-melanocyte-stimulating hormone (α-MSH), a marker for the hypothalamus and pituitary gland^14^. We detected *spx1* expression in the midbrain and hindbrain (Fig. 1H,I), but not in the hypothalamus (Fig. 1J). In contrast, *spx2*-expressing cells were observed in the preoptic area of anterior hypothalamus (Fig. 1K), rather than a-MSH expressing hypothalamus or pituitary (Fig. 1L), suggesting that SPX2 may be involved in neuroendocrine function.

**Figure 2:**
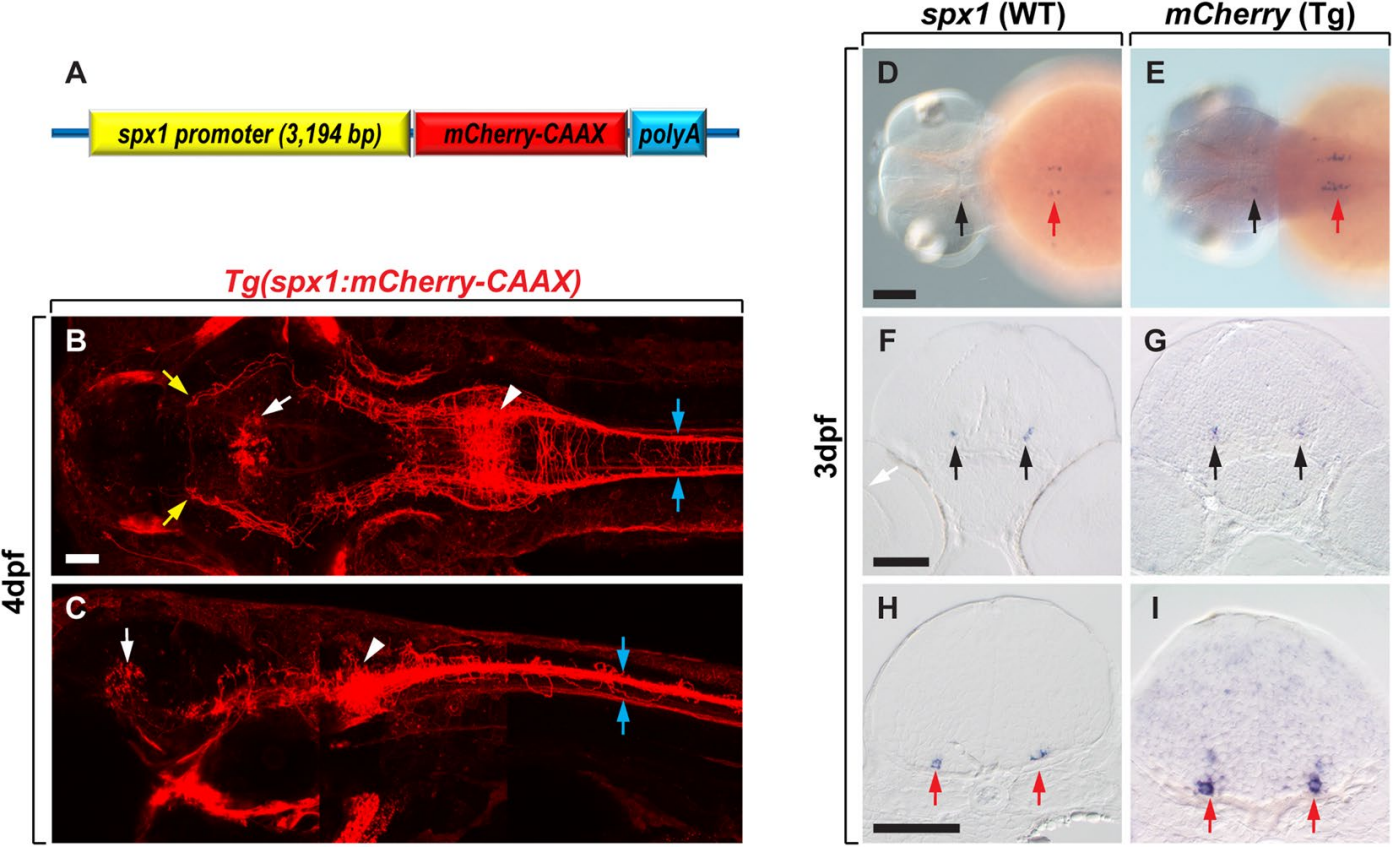
*Tg*(*spx1:mCherry-CAAX*) zebrafish express mCherry-CAAX in *spx1*-expressing cells. (**A**) Schematic of the plasmid construct for the generation of *Tg*(*spx1:mCherry-CAAX*) zebrafish. (**B**,**C**) Dorsal (**B**) and lateral (**C**) views of *Tg*(*spx1:mCherry-CAAX*) zebrafish at 4 days post-fertilisation (dpf), anterior to the left. White arrowheads designate *spx1*-expressing neurons in the hindbrain, and blue arrows indicate *spx1*^+^ axonal projections to the spinal cord. White arrows mark the midbrain tegmentum, and yellow arrows label *spx1*^+^ axonal projections to the forebrain. (**D**–**I**) *In situ* RNA hybridization of 3 dpf wildtype and *Tg*(*spx1:mCherry-CAAX*) embryos with *spx1* (**D**,**F**,**H**) and *mcherry* mRNA (**E**,**G**,**I**), respectively. Dorsal views of whole-mount embryos with anterior to the left (**D**,**E**), and transverse sections of the brain with dorsal to the top (**F**–**I**). Black and red arrows indicate labelled cells in the midbrain tegmentum and hindbrain, respectively. Scale bar: 50 μm in B,C,F–I; 100 μm in D,E.

To investigate the identity and neural circuitry of *spx1*-expressing cells in the zebrafish brain, we isolated ~3 kb of the 5′-upstream region of zebrafish *spx1* from zebrafish genomic DNA by polymerase chain reaction (PCR) and established *Tg*(*spx1:mCherry-CAAX*) and *Tg*(*spx1:gal4*; *uas:egfp*) zebrafish which express mCherry-CAAX and EGFP fluorescent protein, respectively, in *spx1*-expressing neurons under the control of the *spx1* promoter (Fig. 2 and Supplementary Fig. 2). Consistent with *spx1* mRNA expression, mCherry fluorescence was detected in midbrain and caudal hindbrain neurons, and their nerve fibers in *Tg*(*spx1:mCherry-CAAX*) larvae (Fig. 2B,C). We also observed ascending projections from hindbrain SPX1 neurons to the midbrain area, descending projections to the spinal cord, and projections crossing the midline (Fig. 2B, arrows). Whole-mount *in situ* RNA hybridization for *spx1* and *mCherry* mRNA revealed that mCherry was expressed in the midbrain and hindbrain in a pattern similar to that of the endogenous *spx1* gene (Fig. 2D–I), suggesting that mCherry fluorescent protein expression was driven by *spx1* regulatory elements.

**Figure 3:**
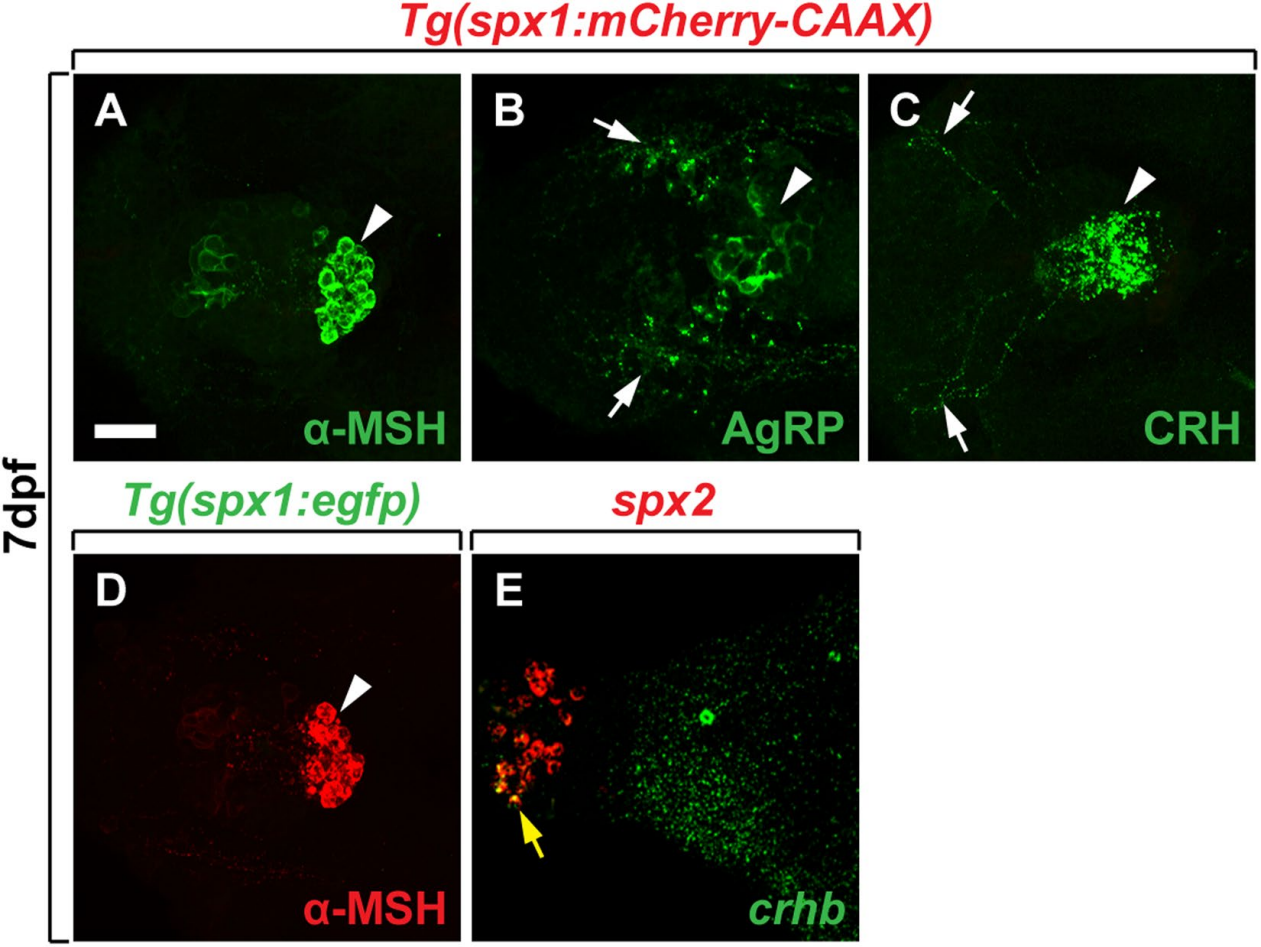
*spx 1* and *spx 2* expression in the hypothalamic area of zebrafish larvae. (**A**–**E**) Labelling of 7 dpf *Tg*(*spx1:mCherry-CAAX*) (**A**–**C**), *Tg*(*spx1:gal4*; *uas:egfp*) (**D**), and wildtype larvae (**E**) with antibodies against α-melanocyte-stimulating hormone (α-MSH) (**A**,**D**), agouti-related peptide (AgRP) (**B**), corticotropin-releasing hormone (CRH) (**C**), and *corticotropin releasing hormone b* (*crhb*) RNA probe (**E**). Ventral views with anterior to the left. White arrowheads indicate α-MSH expression in the pituitary gland (**A**,**D**), and white arrows label AgRP-expressing neurons in the ventral hypothalamus (**B**) and CRH^+^ axons in the hypothalamus (**C**). (**E**) Expression of *spx2* and *crhb* mRNA in the larval brain revealed by *in situ* RNA hybridization. Yellow arrow designates neurons co-expressing *spx2* and *crhb* mRNA in the preoptic region of the hypothalamus. Scale bar: 25 μm.

To confirm SPX1/2 expression in the neuroendocrine system, we next examined mCherry and EGFP expression in *Tg*(*spx1:mCherry-CAAX*) and *Tg*(*spx1:gal4*; *uas:egfp*) larvae using antibodies against α-MSH, agouti-related protein (AgRP)^14^ and corticotropin-releasing hormone (CRH)^15^, markers for the hypothalamus and pituitary gland (Fig. 3). *Tg*(*spx1:mCherry-CAAX*) zebrafish were used to investigate axonal projections because mCherry-CAAX is localized in the membrane. *Tg*(*spx1:gal4*; *uas:egfp*) zebrafish were used to detect SPX1-expressing neuronal cell bodies (Supplementary Fig. 2). To detect *spx2* expression, fluorescent *in situ* RNA hybridization was employed. Consistent with *spx1* mRNA expression, *spx1*:mCherry^+^ and *spx1*:EGFP^+^ cells were not detectable in α-MSH^+^ (Fig. 3A,D), AgRP^+^ (Fig. 3B), or CRH^+^ (Fig. 3C) neurons in the hypothalamus and pituitary gland. Consistent with spx2 mRNA expression revealed by *in situ* RNA hybridization (Fig. 1E–G,K), a few *spx2-*expressing cells were detected in the preoptic area delineated by *crhb* expression (Fig. 3E, arrow).

### Phenotypic and circuit-level characterisation of hindbrain SPX1 neurons

**Figure 4:**
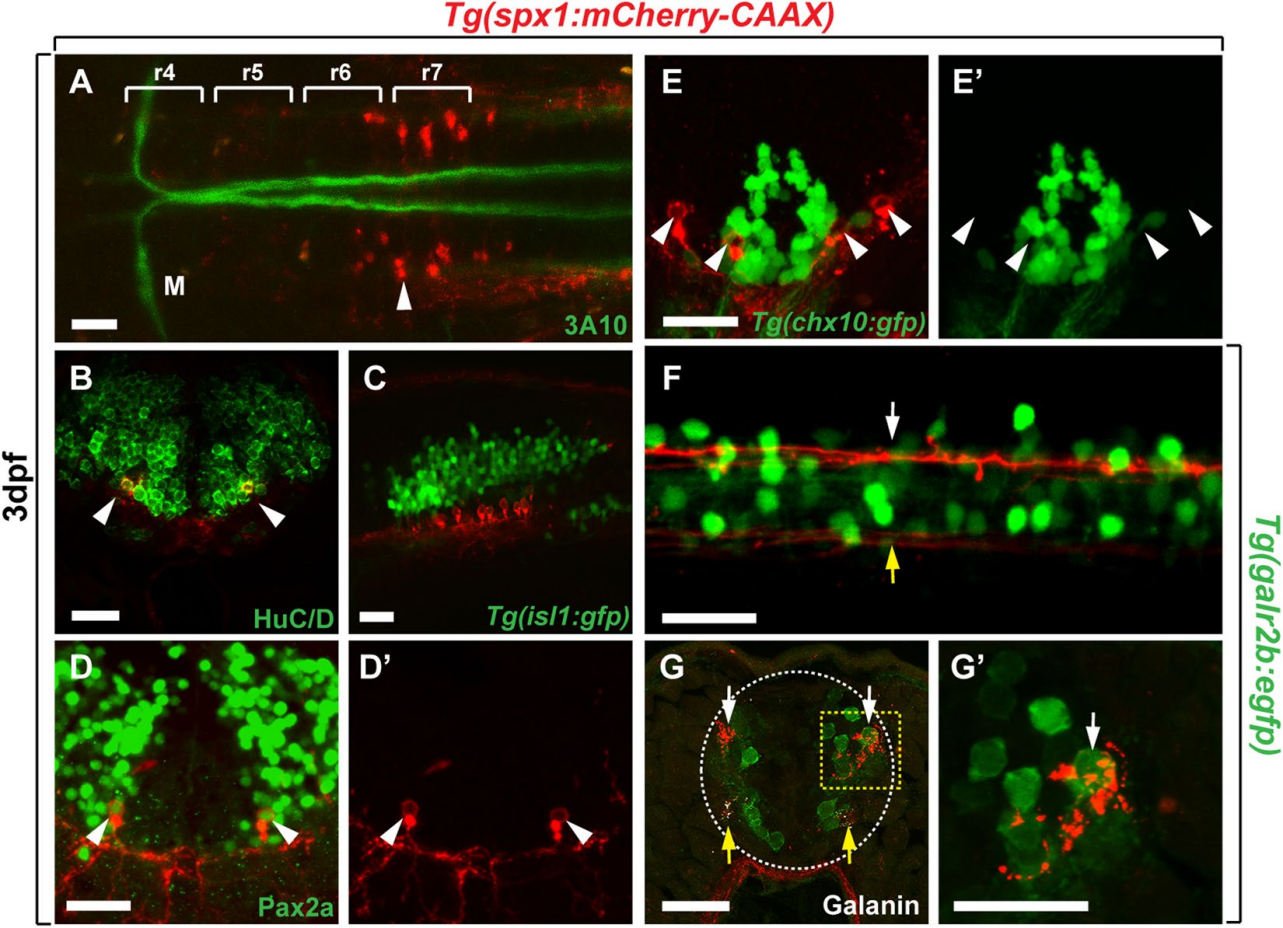
Characterisation of hindbrain SPX1 neurons and their projections. Dorsal (**A**) and lateral (**C**,**F**) views of zebrafish larvae at 3 days post-fertilisation (dpf), anterior to the left. Transverse sections of the hindbrain (**B**,**D**,**D’**,**E**,**E’**) and spinal cord (**G**,**G’**) at 3 dpf, dorsal to the top. (**A**,**B**) Labelling of *Tg*(*spx1:mCherry-CAAX*) larvae with anti-3A10 and anti-HuC/D antibodies to detect Mauthner axons (**A**, green) and post-mitotic neurons (**B**, green), respectively. (**C**) *Tg*(*spx1:mCherry-CAAX*); *Tg*(*isl1:gfp*) larvae to detect hindbrain SPX1 neurons and cranial motor neurons (green). (**D**,**D’**) Labelling of *Tg*(*spx1:mCherry-CAAX*) larvae with anti-Pax2 antibody (green). mCherry-CAAX fluorescence is detected in the membrane surrounding Pax2a^+^ neuronal cell bodies. (**E**,**E’**) Transverse section of the hindbrain of *Tg*(*spx1:mCherry-CAAX*); *Tg*(*chx10:gfp*) larvae. Arrowheads indicate spx:mCherry^+^/chx10:EGFP^−^ neurons. (**F**) Lateral view of the spinal cord of *Tg*(*spx1:mCherry-CAAX*); *Tg*(*galr2b:egfp*) larvae. Arrows indicate spinal projections of hindbrain SPX1 neurons. (**G**,**G’**) Transverse sections of the spinal cord of *Tg*(*spx1:mCherry-CAAX*); *Tg*(*galr2b:egfp*) larvae. White arrows indicate the axonal projections of hindbrain SPX1 neurons, which interact with *galr2b*-expressing neurons in the dorsal spinal cord. Yellow arrows indicate the axonal projections of hindbrain SPX1 neurons in the ventral spinal cord. Abbreviation: M, Mauthner neuron; r, rhombomere. Scale bar: 25 μm.

We next characterised the identity and neural connectivity of SPX1 neurons in the hindbrain. Approximately 20–30 *spx1*:mCherry^+^ cells were observed in the caudal hindbrain of *Tg*(*spx1:mCherry-CAAX*) zebrafish at 3 days post fertilisation (dpf) (Fig. 4A). Labelling of *Tg*(*spx1:mCherry-CAAX*) embryos with an anti-3A10 antibody, which marks Mauthner cells in rhombomere 4 of the hindbrain and their projections to the spinal cord^16^, indicated that *spx1*:mCherry^+^ cells were located in the caudal area of the hindbrain close to rhombomeres 6–7 (Fig. 4A). Labelling with an anti-HuC/D antibody revealed that hindbrain *spx1*:mCherry^+^ cells were HuC/D^+^ post-mitotic neurons (Fig. 4B). To characterise the neuronal subtype of hindbrain *spx1*:mCherry^+^ cells, we used *Tg*(*isl1:gfp*) zebrafish, which express GFP in cranial motor neurons^17^, and an anti-Pax2 antibody, which marks inhibitory interneurons^17–19^. Analysis of the hindbrain of *Tg*(*spx1:mCherry-CAAX*); *Tg*(*isl1:gfp*) larvae revealed that hindbrain *spx1*:mCherry^+^ neurons were located near *isl1*:GFP^+^ cells but were not Islet1^+^ cranial motor neurons (Fig. 4C). Instead, labelling with an anti-Pax2 antibody showed that *spx1*:mCherry^+^ neurons were Pax2a^+^ interneurons (Fig. 4D,D’). To further examine the axonal projections of *spx1*:mCherry^+^ neurons in the hindbrain, we analysed mosaic expression of *spx1*:mCherry in the hindbrain of *spx1*:mCherry DNA-injected zebrafish larvae. We observed that hindbrain *spx1*:mCherry^+^ neurons had T-shaped axonal projections including ascending projections to the midbrain area, descending projections to the spinal cord, and projections crossing the midline (Supplementary Fig. 3).

Chx10-expressing V2a neurons in the hindbrain include reticulospinal neurons which have ipsilateral projections to the spinal cord and play critical roles in locomotor drive^20^. Since hindbrain *spx1*:mCherry^+^ neurons had ipsilateral projections to the spinal cord (Fig. 2B and Supplementary Fig. 3), we next examined whether these neurons were also Chx10^+^ reticulospinal neurons using *Tg*(*chx10:gfp*) zebrafish, which express GFP in Chx10^+^ V2a neurons^21^. Analysis of *Tg*(*spx1:mCherry-CAAX*); *Tg*(*chx10:gfp*) larval hindbrain revealed that *spx1*:mCherry^+^ cells did not colocalise with medially located *chx10*:GFP^+^ V2a neurons. Instead, they were located in the lateral area of the hindbrain, separate to the Chx10^+^ neuronal cluster (Fig. 4E,E’). Zebrafish SPX1 binds to and activates galanin receptor 2a (GALR2a) and 2b (GALR2b), with higher potency toward GALR2b than that of galanin, suggesting that SPX1 is a functional agonist for GALR2b in zebrafish^12^. Therefore, we next examined whether hindbrain SPX1 neurons interact with GALR2b neurons in the spinal cord using *Tg*(*galr2b:egfp*) zebrafish, which express EGFP in *galr2b*-expressing neurons^22^. Analysis of the spinal cord of *Tg*(*spx1:mCherry-CAAX*); *Tg*(*galr2b:egfp*) larvae revealed that hindbrain SPX1 neurons projected to the dorsal and ventral spinal cord, whereby dorsal projections were much thicker than ventral projections in the spinal cord (Fig. 4F,G’, arrows). Immunolabelling of *Tg*(*spx1:mCherry-CAAX*); *Tg*(*galr2b:egfp*) larval spinal cord with an anti-galanin antibody revealed that *spx1*:mCherry^+^ projections passed through the dorsal spinal cord and contacted with *galr2b*:EGFP^+^ neurons where galanin immunoreactivity was not detectable (Fig. 4G,G’). Galanin immunoreactivity was detected only in the white matter of the ventral spinal cord (Fig. 4G, yellow arrows), suggesting that GALR2b neurons in the dorsal spinal cord interact with hindbrain SPX1 neurons but ventral GALR2b neurons interact with galanin and/or SPX1.

**Figure 5:**
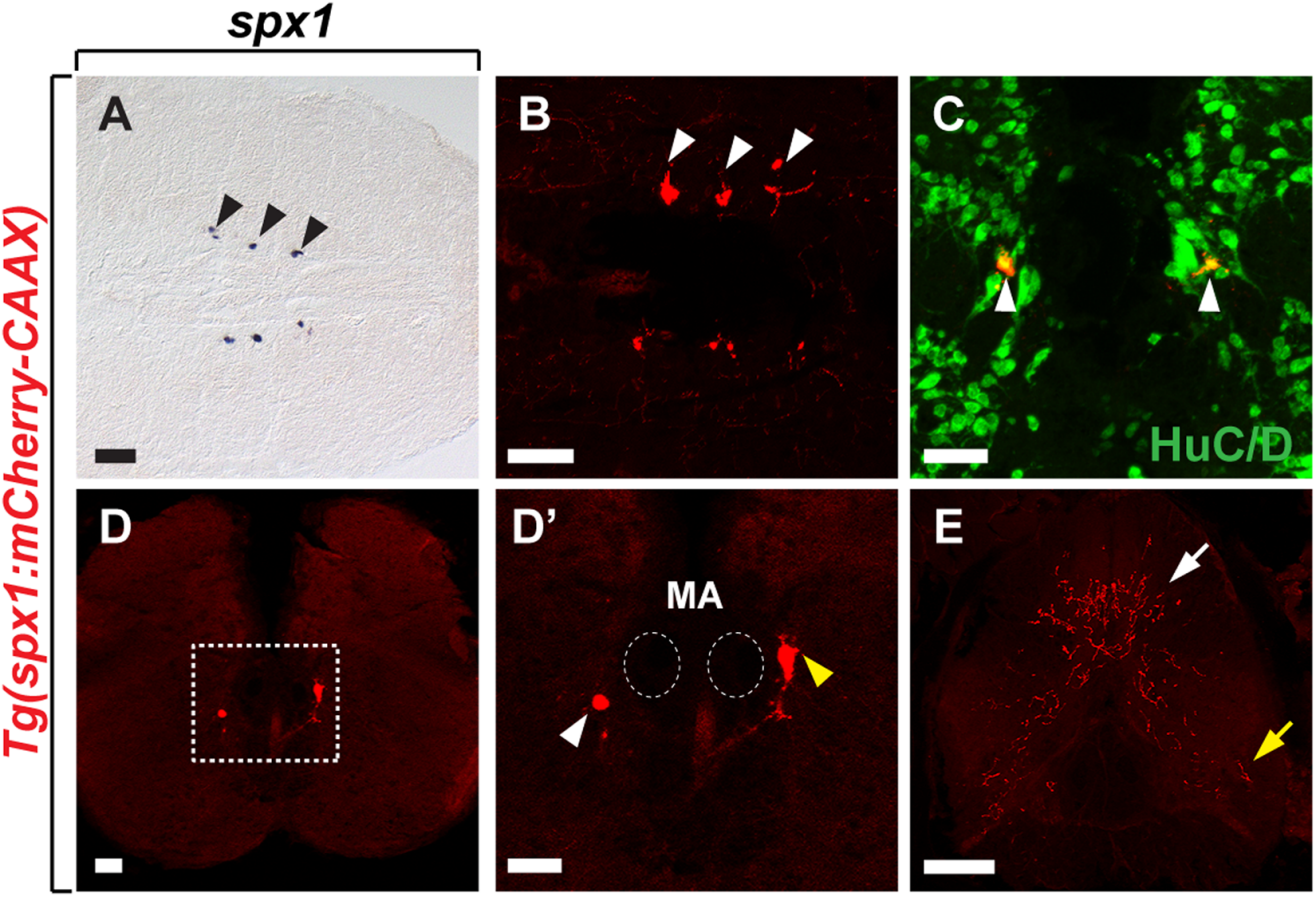
Characterisation of SPX1 neurons in the hindbrain of adult zebrafish. Horizontal (**A**,**B**) and transverse (**C**–**E**) sections of the brain (**A**–**D’**) and spinal cord (**E**) of adult *Tg*(*spx1:mCherry-CAAX*) zebrafish. (**A**,**B**) Anterior is to the left and (**C**–**E**) dorsal is to the top. SPX1 neurons revealed by whole-mount *in situ* RNA hybridization with *spx1* RNA (**A**) and *spx1*:mCherry expression (**B**). (**C**) Labelling of the hindbrain of *Tg*(*spx1:mCherry-CAAX*) adult zebrafish with anti-HuC/D antibody. (**B**,**C**) White arrowheads indicate *spx1*:mCherry neurons in the brainstem. (**D**) *spx1*:mCherry expression is detected in the inferior reticular formation region of the hindbrain. (**D’**) Magnified image of the boxed area in the panel D. Dotted circles mark the position of Mauthner axons in the hindbrain. White and yellow arrowheads indicate the inferior reticular formation region and vagal motor nucleus region, respectively. (**E**) Axons of hindbrain SPX1 neurons projecting to the spinal cord. White and yellow arrows indicate axonal projections in the dorsal and ventral spinal cord, respectively. Abbreviation: MA, Mauthner axon. Scale bar: 50 μm.

We next examined whether *spx1* was continuously expressed in the brainstem of adult zebrafish using whole-mount *in situ* RNA hybridization and confocal imaging of Tg(*spx1:mCherry-CAAX*) zebrafish (Fig. 5). Consistent with SPX1 expression in larvae, *spx1* mRNA (Fig. 5A) and *spx1*:mCherry^+^ expression (Fig. 5B) were detected bilaterally in the brainstem of adult zebrafish. Immunolabelling with an anti-HuC/D antibody revealed that brainstem *spx1*:mCherry^+^ cells are HuC/D^+^ post-mitotic neurons (Fig. 5C). Transverse sections of the brainstem of adult *Tg*(*spx1:mCherry-CAAX*) zebrafish indicated that SPX1 neurons were located in the vagal motor nucleus and inferior reticular formation region (Fig. 5D,D’). We also observed SPX1 neuronal projections throughout the spinal cord of adult *Tg*(*spx1:mCherry-CAAX*) zebrafish, with enriched dorsal projections (Fig. 5E).

**Figure 6:**
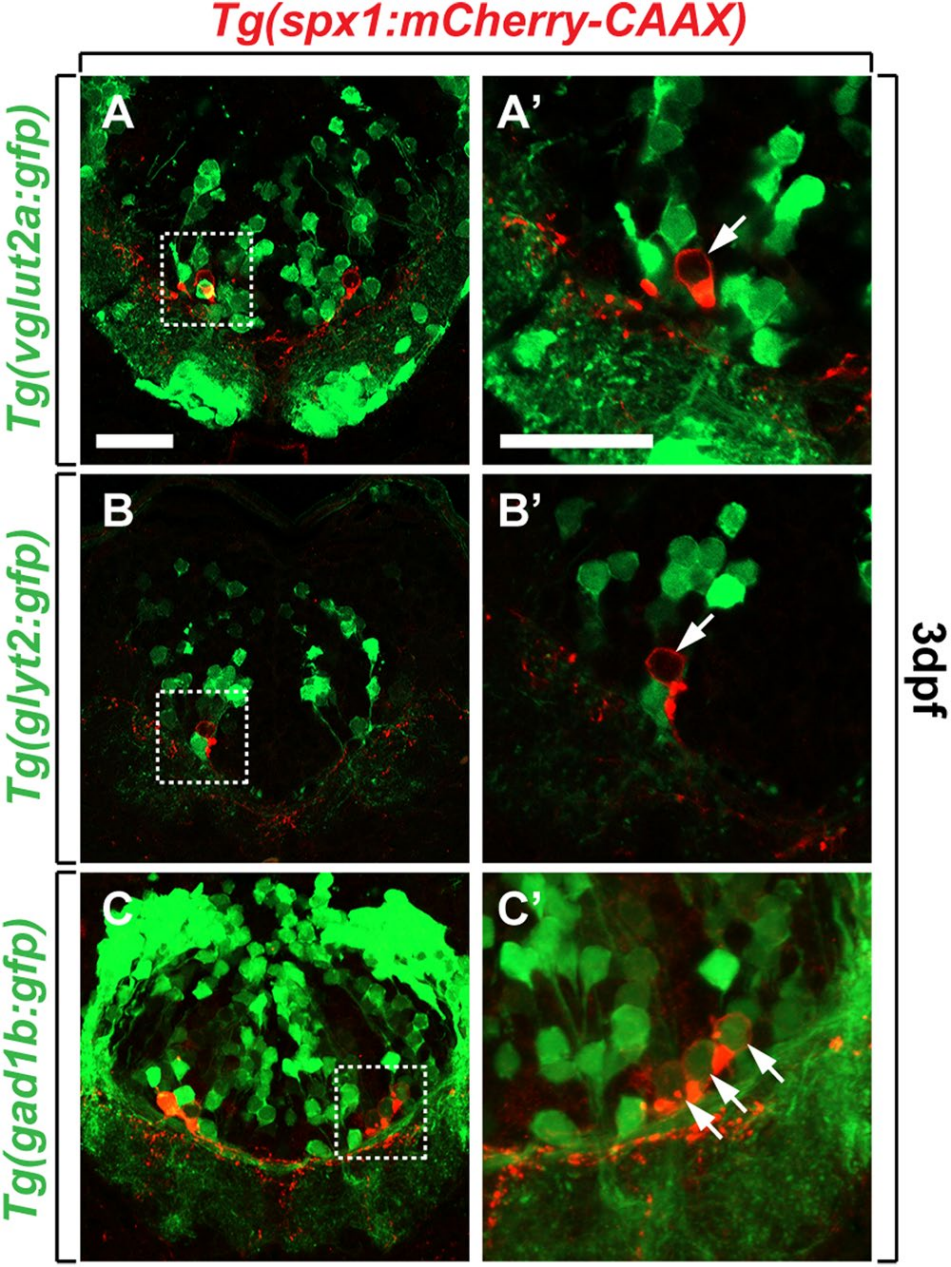
Identification of neurotransmitter phenotype of SPX1 neurons in the hindbrain. (**A**–**C’**) Transverse sections of the hindbrain of *Tg*(*spx1:mCherry-CAAX*); *Tg*(*vglut2a:gfp*) (**A**,**A’**), *Tg*(*spx1:mCherry-CAAX*); *Tg*(*glyt2:gfp*) (**B**,**B’**), and *Tg*(*spx1:mCherry-CAAX*); *Tg*(*gad1b:gfp*) (**C**,**C’**) at 3 days post-fertilisation (dpf), dorsal to the top. Panels labelled with a prime are the magnified images of the boxed areas in each panel. Arrows mark *vglut2a*-negative (**A’**), *glyt2*-negative (**B’**) and *gad1b*-positive (**C’**) *spx1*-expressing neurons in the hindbrain. Scale bar: 25 μm.

We next investigated whether hindbrain SPX1 neurons were excitatory or inhibitory by analysing their neurotransmitter content. Zebrafish hindbrain neurons have been shown to express various neurotransmitters including the vesicular glutamate transporter, *vglut1*, *vglut2a*, and *vglut2b* (markers for excitatory glutamatergic neurons); the neuronal glycine transporter, *glyt2* (marker for inhibitory glycinergic neurons); and glutamic acid decarboxylase, *gad65* (or *gad2*) and *gad67* (or *gad1b*) (markers for inhibitory GABAergic neurons)^23^. To investigate the neurotransmitter phenotype of hindbrain SPX1 neurons, we used *Tg*(*vglut2a:gfp*), *Tg*(*gad1b:gfp*)^24^ and *Tg*(*glyt2:gfp*)^25^ zebrafish which mark glutamatergic, GABAergic, and glycinergic neurons, respectively. Analysis of the hindbrain of double transgenic zebrafish larvae revealed that a subpopulation of *spx1*:mCherry^+^ neurons co-localised with *gad1b*:GFP (Fig. 6C,C’) but not *vglut2a*:GFP^+^ (Fig. 6A,A’) or *glyt2*:GFP^+^ neurons (Fig. 6B,B’). These findings suggested that a subpopulation of hindbrain SPX1 neurons was GABAergic.

### Expression of *spx1* and *galr2a/2b* in the habenula-interpeduncular nucleus circuitry

**Figure 7:**
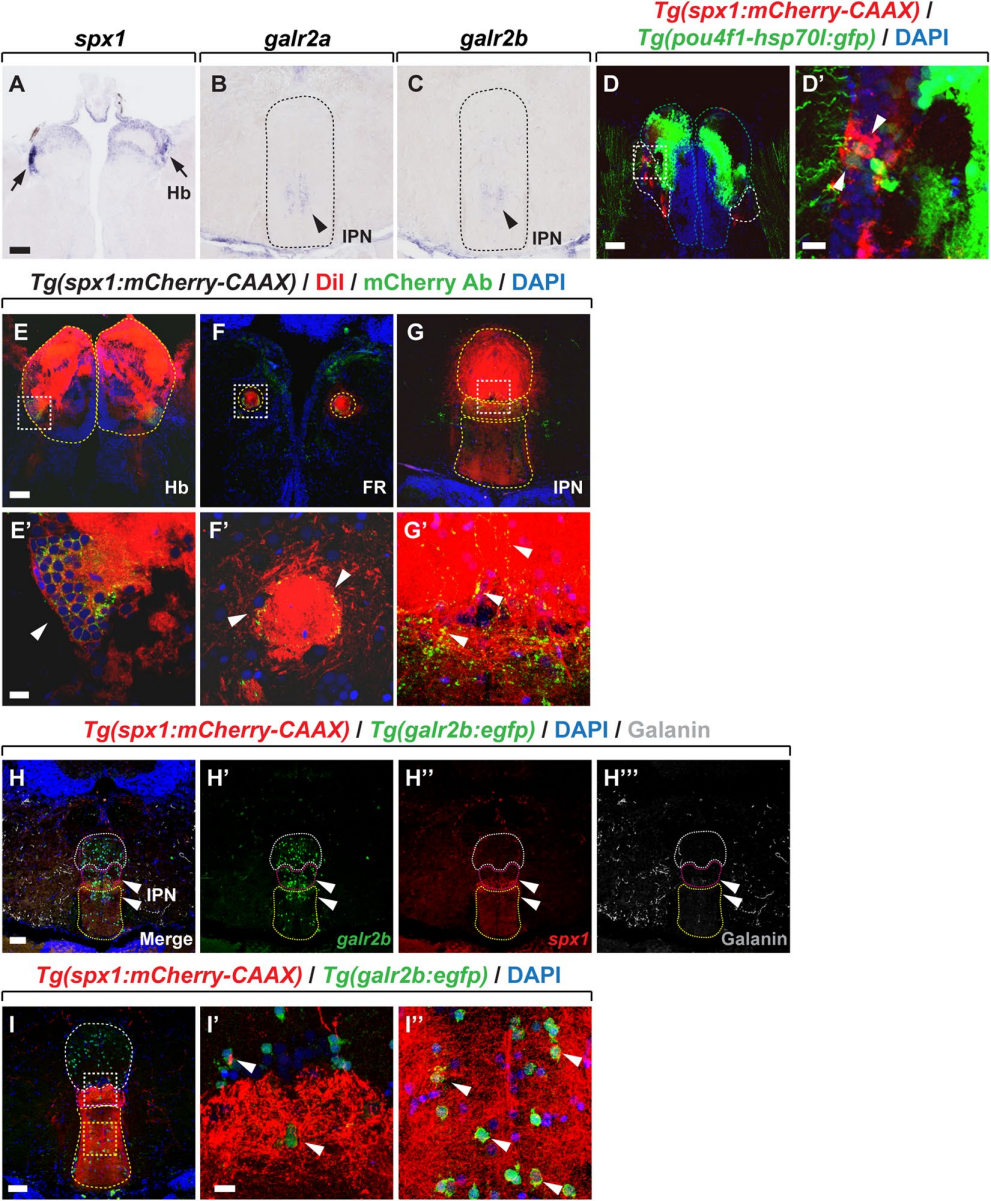
SPX1 and GALR2a/2b expression in the habenula and interpeduncular nucleus. Transverse sections of the adult zebrafish brain, dorsal to the top. (**A**–**C**) *In situ* RNA hybridization in the adult brain with *spx1* (**A**), *galr2a* (**B**), and *galr2b* (**C**) RNA. (**A**) Arrows indicate the expression of *spx1* mRNA in the habenula. Black arrowheads indicate *galr2a* and *galr2b* mRNA expression, respectively, in the IPN (**B**,**C**). (**D**,**D’**) Transverse sections of the habenula (**D**) of *Tg*(*spx1:mCherry-CAAX*); *Tg*(*pou4f1-hsp70l:gfp*) zebrafish. (**D**) White, green, and blue dotted lines mark lateral and medial subdivisions of the dorsal habenula and ventral habenula, respectively. (**D’**) is a high magnification image of the box area in (**D**). White arrowheads indicate *pou4fl*^+^ *spx1*:mCherry-expressing cell body in the dHbM. (**E**–**G’**) Transverse section of the Hb (**E**,**E’**), FR (**F**,**F’**), and IPN (**G**,**G’**) of DiI-labelled *Tg*(*spx1:mCherry-CAAX*) zebrafish. Panel E’–G’ are high magnification images of white boxes in panel E–G. Yellow dotted lines mark DiI-labelled Hb (**E**), FR (**F**), and IPN (**G**). White arrowheads indicate that co-localization of DiI and mCherry fluorescence. (**H**–**I”**) Transverse sections of *Tg*(*spx1:mCherry-CAAX*); *Tg*(*galr2b:egfp*) zebrafish, which were labelled with DAPI. White, magenta, and yellow dotted lines mark the dorsal, intermediate, and ventral IPN, respectively. (**H**–**H”’**) Section views labelled with anti-galanin antibody. White arrowheads indicate the intermediate and ventral IPN, where *spx1*:mCherry^+^ axonal projections were observed. (**I**–**I”**) Panel I’ and I” are high magnification images of white and yellow boxes of panel I, respectively. White arrowheads indicate close contacts between EGFP^+^ cells and mCherry^+^ nerve fibres. Abbreviation: FR, fasciculus retroflexus; Hb, habenula; IPN, interpeduncular nucleus; Scale bar: 50 μm in A–I. 10 μm in D’–I”.

*Galr2* and *Galr3* knockout mice exhibit anxiety and depression-like phenotypes^26–28^. Furthermore, GALR2 and GALR3 have been shown to be putative receptors for SPX *in vitro*^12^, suggesting that SPX and GALR2/3 interaction may be involved in the regulation of anxiety and mood disorders. The medial habenula-interpeduncular nucleus (IPN) circuitry is implicated in anxiety and mood regulation. We thus investigated the expression of *spx1*/2, *galr2a*/2b, and galanin in the habenula and IPN. Whole-mount *in situ* RNA hybridization of the adult zebrafish brain for *spx1*, *spx2*, *galr2a*, and *galr2b* mRNA revealed that only *spx1* was expressed in the dorsal habenula, which is the homologue of the medial habenula in mice (Fig. 7A, arrows), and *galr2a/2b* expression was detected in the ventral area of the IPN (Fig. 7B,C, arrowheads). To investigate in more detail the expression of SPX1/2 and GALR2a/2b in these regions, we used *Tg*(*pou4f1-hsp70l:gfp*) zebrafish, which express GFP in the medial subdivisions of the dorsal habenula (dHbM), which projects to the intermediate and ventral IPN^29^. Consistent with the mRNA expression pattern, *spx1*:mCherry^+^ cells were first detected in a subpopulation of dorsal habenula neurons in 7 dpf larvae (Supplementary Fig. 4A) and its expression was also detected in adult *Tg*(*spx1:mCherry-CAAX*); *Tg*(*pou4f1-hsp70l:gfp*) zebrafish. However, only a few *spx1*:mCherry^+^ cells were co-localized with GFP^+^ cells (Fig. 7D,D’). Fluorescent *in situ* RNA hybridization with *spx1* in the adult brain of *Tg*(*narp:gal4vp16*); *Tg*(*uas:dsRed*) zebrafish, which expresses DsRed in the lateral division of the dorsal habenula (dHbL), indicated that parts of *spx1*:mCherry^+^ cells were located in the dHbL (Supplementary Fig. 4B,B’). Notably, *spx1*:mCherry^+^ nerve fibers were detected in the dorsal IPN (dIPN), intermediate IPN (iIPN) and ventral IPN (vIPN) (Fig. 7G’,H”,I), suggesting that the IPN may receive input from *spx1*:mCherry^+^ cells in the dHb.

To examine whether SPX1 neurons in the Hb project to the IPN, we performed anterograde labelling in the habenula of adult *Tg*(*spx1:mCherry-CAAX*) zebrafish using neuronal tracer, DiI. We first confirmed that habenula cells were labeled with DiI (Fig. 7E) and found that dHbL cells co-localized with DiI and *spx1*:mCherry fluorescence (Fig. 7E’). In addition, we found that *spx1*:mCherry^+^ nerve fibers were co-localized with DiI-positive axon fibers in the fasciculus retroflexus (Fig. 7F,F’) and IPN (Fig. 7G,G’), suggesting that habenular SPX1 neurons project axons to the IPN. To further investigate the expression of galanin, SPX1, and GALR2b in the IPN, we labelled the brain of adult *Tg*(*spx1:mCherry-CAAX*); *Tg*(*galr2b:egfp*) zebrafish with an anti-galanin antibody. We detected *galr2b:*EGFP^+^ neurons throughout the entire IPN. *spx1*:mCherry^+^ nerve fibers were highly enriched in the i/vIPN but weak *spx1*:mCherry^+^ fluorescence was also detected in the dIPN. However, galanin immunoreactivity was rarely observed in the IPN region (Fig. 7H”’), suggesting that GALR2a and GALR2b cells located in the IPN may interact with SPX1 projections from the dHb. A high magnification confocal image of the IPN of adult *Tg*(*spx1:mCherry-CAAX*); *Tg*(*galr2b:egfp*) zebrafish showed that *galr2b:*EGFP^+^ neurons were in close contact with *spx1*:mCherry^+^ nerve fibers (Fig. 7I–I”). Collectively, these data suggest that nerve fibers from dHb SPX1 neurons interact with GALR2b neurons in the IPN, and SPX1-GALR2b interaction in the dHb-IPN circuitry may be involved in anxiety and mood regulation.

## Discussion

In the present study, we describe the expression of SPX1 and SPX2 neuropeptides. We identify the phenotype and connectivity of SPX1 neurons and their interaction with GALR2b neurons in the zebrafish CNS. In mammals, SPX expression is widely expressed in various tissues including brain, skin, respiratory, digestive, urinary, and reproductive systems^1–3^. In teleost, SPX1 is expressed mainly in the brain. The highest level of SPX1 expression was detected by RT-PCR in the goldfish brain including optic tectum, hypothalamus, and brainstem, with lower expression in the cerebellum, telencephalon, pituitary, liver, spleen, heart, intestine, and gonads^5,6^. Zebrafish *spx1* has mainly been detected in the brain and ovary by real-time PCR^5^ but *spx2* expression has yet to be identified. In contrast to the broad expression of SPX in mammals, our data obtained by whole-mount *in situ* RNA hybridization and analysis of transgenic reporter zebrafish indicate that zebrafish *spx1/2* expression is restricted in certain brain regions; *spx1* in the midbrain and brainstem, and *spx2* in the preoptic area of the hypothalamus. We did not detect *spx1* expression in the hypothalamus, where SPX function is implicated in the regulation of reproduction and feeding control^5,6,11^. Thus, SPX2 function may be involved in reproduction and feeding control in zebrafish. However, a recent study reported that *spx1* knock-out zebrafish exhibited normal reproductive capability but higher food intake than wildtype fish, an effect mediated via increased expression of the appetite stimulant AgRP1^11^. These findings suggest that SPX1 is involved in the control of feeding. Since SPX1 expression was not detectable in the hypothalamus, it appears that SPX1 neurons are not directly connected to appetite-regulating AgRP1 neurons, which are in the hypothalamus. We think that SPX1 neurons may be involved in the control the AgRP1 expression indirectly via other neural circuits.

We also observed approximately 20–30 SPX1 neurons bilaterally located in the caudal hindbrain of larval zebrafish and reticular formation region in the brainstem of adult zebrafish. Hindbrain SPX neurons are located in the caudal area near rhombomere 6–7 and have descending projections to the spinal cord, which are the characteristics of reticulospinal neurons. Reticulospinal neurons in the hindbrain play critical roles in providing descending excitatory input to the spinal cord locomotor systems in vertebrates^13,20,30,31^. In zebrafish, sets of serially homologous reticulospinal neurons project to caudal levels of the spinal cord^32^, and T reticulospinal interneurons are located in the caudal area of the hindbrain with T-shaped axons including spinal projections^33^. Based on the location in the caudal hindbrain and T-shaped axonal morphology, we speculated that hindbrain SPX1 neurons may be T reticulospinal interneurons. However, the axonal features of hindbrain SPX1 neurons differ slightly from T reticulospinal interneurons. In contrast to the spinal projections of T reticulospinal neurons which merge with Mauthner axons at the midline^33^, nerve fibers of the hindbrain SPX1 neurons traversed the dorsolateral margin of the hindbrain and formed commissures in the spinal cord midline. Chx10^+^ V2a interneurons in the hindbrain include glutamatergic reticulospinal neurons which provide excitatory input for locomotor drive^20^. However, hindbrain SPX1 neurons did not express Chx10, and they were GABAergic, suggesting that they provide inhibitory input to spinal cord neurons. These data indicate that although hindbrain SPX1 neurons have the phenotype of reticulospinal neurons which are located in the hindbrain and have spinal projections, they have different features to those of previously identified reticulospinal neurons. In addition, we observed that hindbrain SPX1 neurons interacted with dorsally located GALR2b neurons, which did not interact with galanin, in the spinal cord. Compared to galanin, SPX1 has greater potency for GALR2b *in vitro*^12^. Collectively, our data suggest that SPX1 neurons in the hindbrain are reticulospinal neurons with spinal projections, and provide GABAergic inhibitory input for locomotor drive partly via interactions with GALR2b in the spinal cord. Consistent with our data, GABAergic reticulospinal interneurons have been reported to stop swimming in hatchling frog tadpoles by providing inhibitory input to locomotion^34^.

The habenula is critical for behavioural choice and is involved in the regulation of pain, stress, fear/anxiety, sleep, and reward^35^. The habenula consists of the medial and lateral habenula in mammals. The medial habenula mainly projects to the IPN, whereas the lateral habenula mainly projects to the ventral tegmental area and raphé^36^. The IPN is a target of medial habenula neurons and projects to the raphé, which is part of the serotonergic system^37^. The medial habenula is a component of the evolutionarily conserved dorsal diencephalic conduction system, which is essential for anxiety and mood regulation^38,39^. In zebrafish, the dorsal habenula is the orthologue of the medial habenula in mice^40^. Dorsal habenula-IPN circuitry is also implicated in fear and anxiety responses^41,42^. In this study, we found that SPX1, but not SPX2, was expressed in the dHb in adult zebrafish, and its neuronal projections were connected to the IPN. Notably, we also found that *galr2a* and *galr2b* were expressed in the IPN, but galanin immunoreactivity was rarely detected in the habenula and IPN, suggesting that habenula SPX1 neurons may interact with GALR2a or GALR2b neurons in the IPN, and SPX1-GALR2a/2b neuronal circuits may be involved in regulating fear/anxiety responses. Our hypothesis is supported by previous studies, showing that GALR2 and GALR3 null mutant mice exhibit anxiety and depression-like phenotypes^26–28^, while administration of SPX-based GALR2-specific agonist exerts an anxiolytic effect in mice^43^. In addition, phylogeny and synteny analysis of the gene family has shown that zebrafish GALR2 is subdivided into GALR2a and GALR2b but it lacks GALR3, and zebrafish SPX1 can activate GALR2a and GALR2b *in vitro* whereby SPX1 has higher potency toward GALR2b than that of galanin^12^. Collectively, our data suggest that, rather than galanin-GALR2a/2b interactions, habenula SPX1 neurons may interact with GALR2a or GALR2b in the IPN, and SPX1-GALR2a/2b neuronal circuits might be involved in the regulation of fear/anxiety responses.

## Methods

### Zebrafish lines

All experimental procedures were approved by the Korea University Institutional Animal Care& Use Committee (IACUC) and performed in accordance with Animal experiment guidelines of Korea National Veterinary Research and Quarantine Service. Wild-type AB strain zebrafish, *Tg*(*spx1:mCherry-CAAX*), *Tg*(*spx1:gal4*), *Tg*(*uas:egfp*), *Tg*(*galr2b:egfp*)^22^, *Tg*(*isl1:gfp*)^17^, *Tg*(*chx10:gfp*)^21^, *Tg*(*vglut2a:gfp*), *Tg*(*gad1b:gfp*)^24^, *Tg*(*glyt2a:gfp*)^25^, *Tg*(*pou4f1-hsp70l:gfp*)^29^, and *Tg*(*narp:gal4vp16*)^41^, *Tg*(*uas:dsred*)^41^ zebrafish were used in this study. Adult zebrafish and zebrafish embryos were raised in 14 h light and 10 h dark cycle at 28.5 °C. Zebrafish embryos were staged according to days post-fertilisation (dpf), months post-fertilisation (mpf), and morphological characteristics. At 1 dpf, 0.003% (w/v) 1-phenyl-2-thiourea (PTU) in embryo medium was used for blocking pigmentation in zebrafish embryos. Also, 3~6-month-old male and female zebrafish were used in this study.

### Cloning of *spx1*, *spx2*, *galr2a*, and *galr2b*

Total RNA was extracted using TRIzol reagent (Invitrogen) from wild-type strain AB embryos. The reverse transcription of RNA into cDNA was performed using ImProm-II™ Reverse Transcriptase (Promega), according to manufacturer’s instructions. To clone *spx1*, *spx2*, *galr2a*, *galr2b*, and *corticotropin releasing hormone b* (*crhb*), we designed PCR primers using sequence from a GenBank (*spx1*: XM_005164774.1, *spx2*: XM_005162991.1, *galr2a*: XM_002664007.3, *galr2b*: XM_001339133.3, *crhb*: NM_001007379.1) and amplified a product from 1 or 5 dpf cDNA. PCR products were cloned using pGEM-T easy vector (Promega).

### Whole-mount *in situ* RNA hybridization and immunohistochemistry

Since SPX1/2 expression in larval brain and spinal cord (from 3 dpf to 7 dpf) is identical, we analysed the identity and circuit of SPX1 neurons in *spx1:mCherry-CAAX* transgenic fish at 3–4 dpf to obtain a whole brain image. For the analysis of SPX1/2 expression in the neuroendocrine system by *in situ* RNA hybridization and immunohistochemistry, we first isolated the whole brain from the skull of a 7 dpf larvae to improve penetration of probes/antibodies and to get the high-quality images. We used adult brain for the analysis of SPX1/2 expression in habenula because the expression of SPX1/2 in habenula is well detected in adult brain and not in the larval brain. For antisense RNA probe synthesis, the pGEM-T easy vector (Promega) containing *spx1*, *spx2*, *galr2a*, *galr2b*, *crhb*, *gfp*, and *mCherry* were linearised and transcribed by restriction endonucleases (NEB, New England Biolabs) and digoxigenin (DIG) labelling mixture (Roche). Wild-type AB and Tg(*spx1:mCherry-CAAX*) were fixed using 4% formaldehyde solution. *spx1*, *spx2*, *galr2a*, *galr2b*, *crhb*, *gfp*, and *mCherry* transcripts were detected in fixed zebrafish embryos and adult brains by whole-mount *in situ* RNA hybridization. Whole-mount *in situ* RNA hybridization was performed as described previously^44^. For Immunohistochemistry, embryos and adult zebrafish were anesthetized by 200 mg/L of ethyl 3-aminobenzoate methanesulfonate salt (MS222, Sigma) until movement ceased and were then fixed in 4% paraformaldehyde. Fixed embryos were embedded in 1.5% agar blocks containing 5% sucrose and equilibrated in 30% sucrose solution. Frozen blocks were sliced into 10-µm sections using a cryostat microtome and mounted on glass slides. Sections were rinsed with PBS several times and then blocked in 2% bovine serum albumin with sheep serum. After the blocking reaction, sections were treated with primary antibodies for 2 hours at room temperature, washed for 2 hours with phosphate buffered saline (PBS), then treated with the appropriate secondary antibodies for 2 hours at room temperature. Images were obtained from the sections of *Tg*(*spx1:mCherry*-*CAAX*) and *Tg*(*galr2b:egfp*) zebrafish embryos. For immunohistochemistry, we used the following antibodies: mouse anti-HuC/D (1:200, Molecular Probes, Cat. No. A21271), mouse anti-3A10 (1:500, Developmental Studies Hybridoma Bank), rabbit anti-GAL (1:5000, Chemicon, Cat. No. AB5909), rabbit anti-alpha MSH (1:200, Phoenix Pharmaceuticals, INC, Cat. No. H-043-01), rabbit anti-AgRP (1:200, Phoenix Pharmaceuticals, INC, Cat. No. H-003-53), rabbit anti-CRH/CRF (1:500, Advanced Targeting Systems, Cat. No. AB-02), rabbit anti-Pax2 (1:200, Covance, Cat. No. PRB-276P), mouse anti-mCherry/Dsred (1:500, Clontech, Cat. No. 632392), rabbit anti-mCherry/Dsred (1:500, Clontech, Cat. No. 632496) and chicken anti-GFP (1:200, Abcam, Cat. No. AB13970). Alexa Fluor 488- and 568-conjugated secondary antibodies were used for fluorescent detection of primary antibodies (1:1000, Invitrogen, Cat. No. A-11001, A-11004, A11008, A-11011 and Abcam, Cat. No. ab96947).

### Plasmid constructs and generation of transgenic zebrafish lines

To produce *Tg*(*spx1:mCherry-CAAX*) and *Tg*(*spx1:gal4*) zebrafish lines, approximately 3.2 kb fragment of zebrafish genomic DNA containing upstream sequences from the *spx1* ATG start codon, was cloned into p5E-MCS^45^. The following primer set was used for the PCR: forward primer (5′-CCCGACAGCTTTGCAGTATT-3′) and reverse primer (5′-AGCGGCAGGTTCCTCTTCAG-3′). The putative regulatory region was confirmed by sequence analysis. Next, we performed LR reaction with *spx1*-5′-, *mCherryCAAX*-middle- or *gal4*-middle- and *polyA*-3′-entry clones to generate *spx1:mCherry-CAAX* and *spx1:gal4* DNA using LR II clonase (Invitrogen). To establish stable transgenic zebrafish lines, the *spx1:mCherry-CAAX* or *spx1:gal4* DNA were injected into fertilised one-cell stage zebrafish embryos. To screen for germ-line transmitted transgenic zebrafish, we mated *spx1:mCherry-CAAX* DNA-injected founder zebrafish with wild-type zebrafish and screened the progeny for mCherry expression. We mated *spx1:gal4* DNA-injected founder zebrafish with *Tg*(*uas:egfp*) zebrafish and screened the progeny for EGFP expression. The obtained transgenic founder fish were crossed with wild-type zebrafish, and F1 transgenic zebrafish were raised to adulthood.

### DiI Labelling

We used the lipophilic tracer 1, 1′-Dioctadecyl-3,3,3′,3′-Tetramethylindocarbocyanine Perchlorate (DiI, Invitrogen, D282) for neuronal tracing, similar to previous studies^29,46^. We anesthetized the 6-12-month adult zebrafish by MS222 (Sigma) and performed transcardiac perfusion using PBS. The adult fish were then fixed in 4% paraformaldehyde with 0.25% glutaraldehyde (Sigma, G5882). Tiny crystals of the DiI were placed on the habenula of the fixed brains using a fine glass needle. The brains were rinsed by the identical fixative to detach extra dye particles, thereafter, the brains were incubated in the fixative at 37 °C for 3 days. Subsequently, the brains were embedded in 1.5% agar blocks containing 5% sucrose and equilibrated in 30% sucrose solution, followed by immunohistochemistry.

## Supplementary information

Supplementary Information

## Data Availability

Data supporting the findings of this study are available in the article and its Supplementary Information Files, or from the corresponding authors on reasonable request.
