# Supplementary material for: Distribution and neuronal circuit of spexin 1/2 neurons in the zebrafish CNS
Source: Sci Rep. 2019 Mar 22;9:5025. doi: 10.1038/s41598-019-41431-7 (PMC6430828; doi:10.1038/s41598-019-41431-7)
Supplement: Supplementary file 1 — Supplementary Information [file 41598_2019_41431_MOESM1_ESM.pdf]

Distribution and neuronal circuit of spexin 1/2 neurons in the zebrafish CNS

Eunmi Kim[†], Inyoung Jeong[†], Ah-young Chung, Suhyun Kim, Seung-Hae Kwon[1], Jae Young Seong[2] and Hae-Chul Park*

Department of Biomedical Sciences, Korea University, Ansan, Gyeonggido 425-707, Republic of Korea; [1]Korea Basic Science Institute, Chun-Cheon, Gangwon-Do 24341, Republic of Korea; [2]Department of Biomedical Sciences, Korea University, Seoul 136-705, Republic of Korea

[†] These authors contributed equally to this work

***Correspondence to**: Hae-Chul Park, Department of Biomedical Sciences, Korea University, Ansan, Gyeonggido 425-707, Republic of Korea.

Telephone: +82-31-412-6713

Fax: +82-31-412-6729

Email: hcpark67@korea.ac.kr.

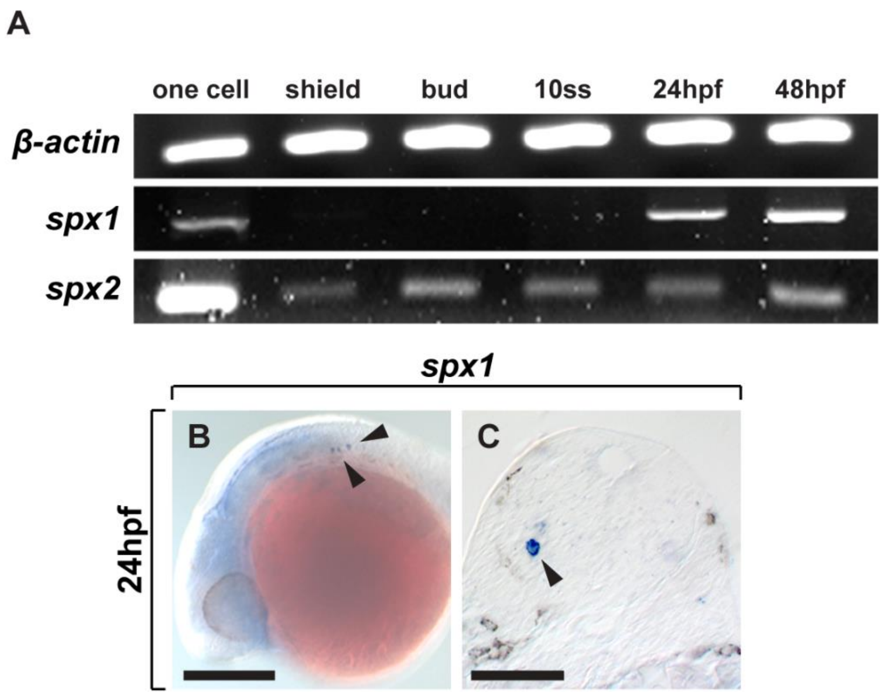

Supplementary Figure 1. Reverse transcriptase PCR (RT-PCR) and *in situ* RNA hybridization of *spx1* and *spx2* during early developmental stages in zebrafish. (A) RT-PCR of *spx1* and *spx2*. cDNA from early developmental stages were used. *β-actin* was used as a control gene for RT-PCR. (B) *In situ* RNA hybridization using *spx1* anti-sense RNA probe at 24 hpf. Lateral view of the brain and anterior is to the left. Arrowheads indicate the expression of *spx1* mRNA in the hindbrain. (C) Transverse sections of the brain in (B) and dorsal is to the top. Arrowhead indicates the *spx1*-expressing cell in the hindbrain. Abbreviation: -ss, somite-stage. Scale bar: 100 μm in B and C.

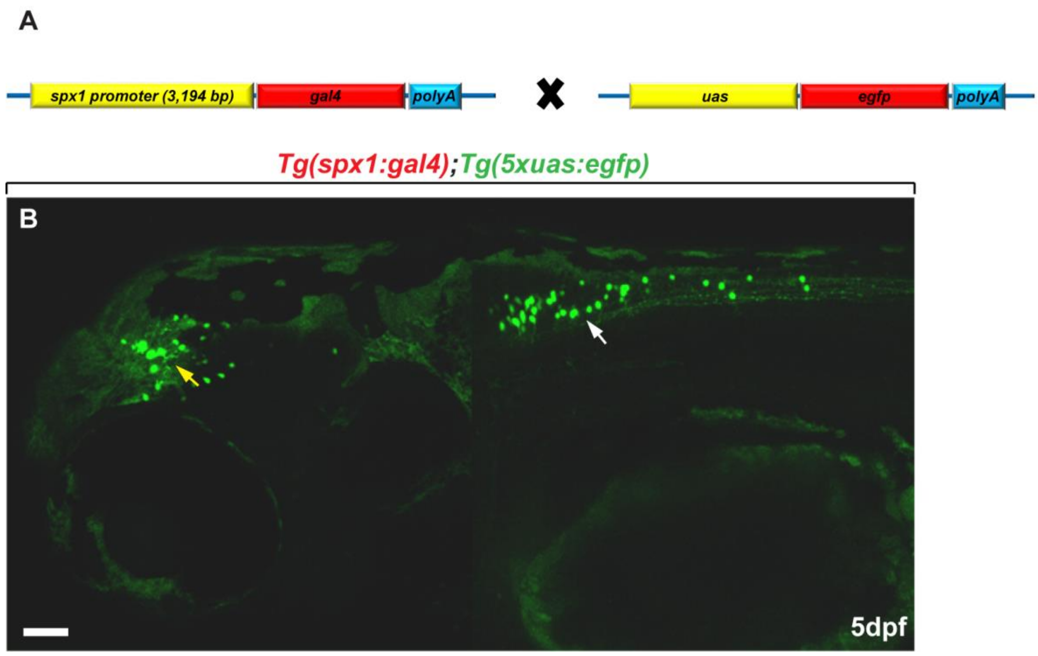

Supplementary Figure 2. Generation of *Tg(spx1:gal4);Tg(uas:egfp)* zebrafish. (A) Schematic of the plasmid construct. (B) Lateral view of *Tg(spx1:gal4);Tg(uas:egfp)* zebrafish at 5 days post fertilisation (dpf). Yellow and white arrows indicate spx1-expressing neurons in the midbrain and hindbrain, respectively. Scale bar: 50 µm.

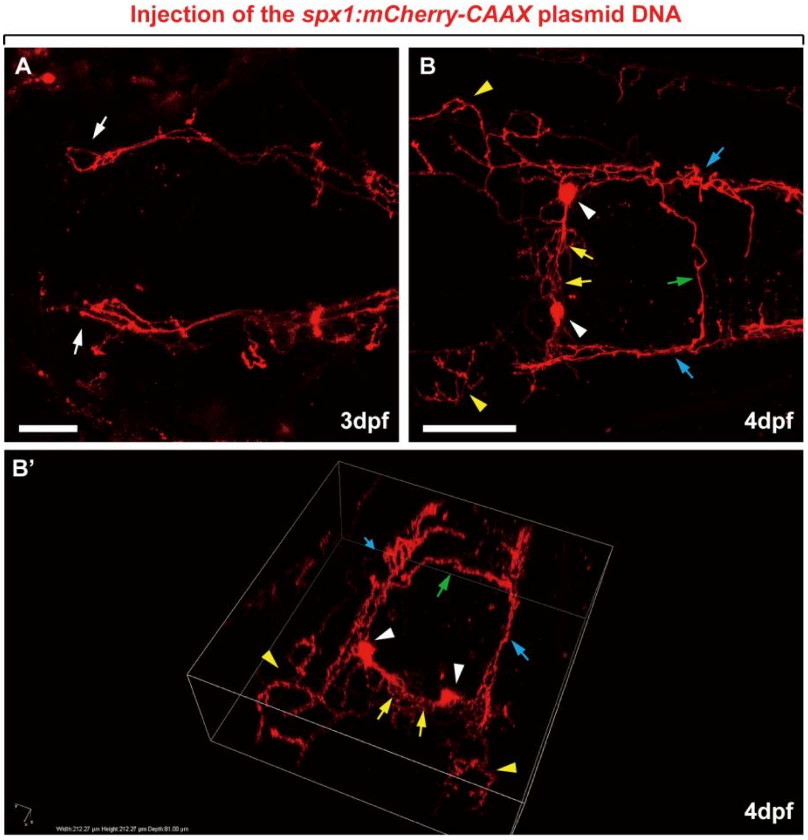

Supplementary Figure 3. Mosaic analysis of the axonal projections of hindbrain spx1^{+} neurons. (A-B) Dorsal views of the hindbrain of 2 days post fertilisation (dpf) (A) and 3 dpf (B, B’) zebrafish larvae injected with *spx1:*mCherry-CAAX recombinant DNA, anterior to the left. (A) White arrows indicate the axonal projections of *spx1*-expressing hindbrain neurons in the forebrain and midbrain. (B-B’) Panel B’ is the three-dimensional rendering image of panel B. White arrowheads mark *spx1*-expressing neuronal cell bodies in the hindbrain. Yellow arrows designate axonal projections to the midline from hindbrain *spx1:*mCherry^{+} neurons. Yellow arrowheads label the ascending projections to the forebrain and midbrain. Blue and green arrows mark descending projections to the spinal cord and the projections crossing the midline, respectively. (B-B’) Direction X: anterior-side, Y: right-side, Z: ventral-side. White box size: Width: 212.27 µm, height: 212.27 µm, depth: 81.00 µm. Scale bar: 50 µm.

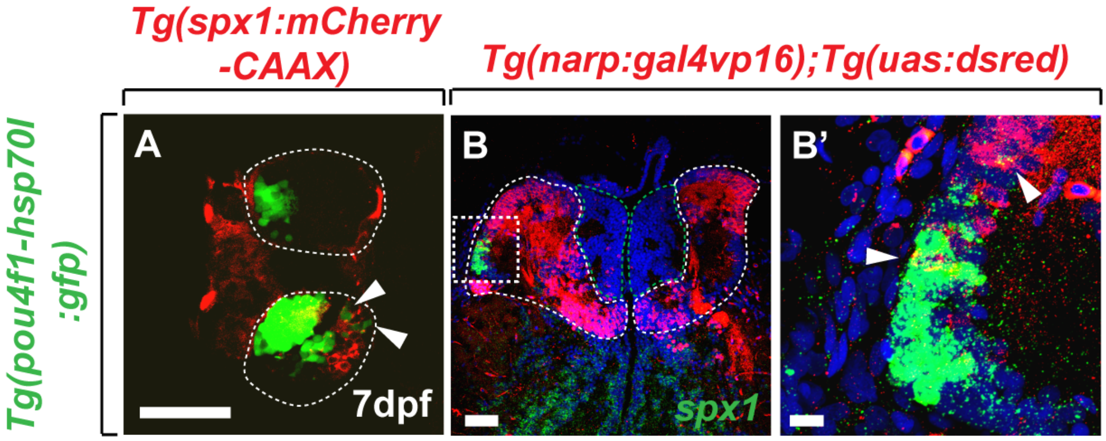

Supplementary Figure 4. Expression of *spx1* in dorsal Hb.

(A) Dorsal view of the Hb region in *Tg(spx1:mCherry-CAAX);Tg(pou4fl-hsp70l:gfp)* larva at 7 dpf. Arrowheads indicate the *spx1* and *pou4fl*$^{+}$ cells in Hb. (B, B') Transverse section of the Hb in *Tg(narp:gal4vp16);Tg(uas:dsred)* adult brain labelled by *spx1* RNA and dorsal is to the top. (B') is a high magnification image of the box area in (B). Arrowhead indicates the *spx1* and *narp*$^{+}$ cells in lateral division of the dorsal Hb. Abbreviation: Hb, habenula; IPN, interpeduncular nucleus. Scale bar: 50 µm in A and B. 10 µm in B'.
